# Dysfunctional Freezing Responses to Approaching Stimuli in Persons with a Looming Cognitive Style for Physical Threats

**DOI:** 10.3389/fpsyg.2016.00521

**Published:** 2016-04-19

**Authors:** John H. Riskind, Laura Sagliano, Luigi Trojano, Massimiliano Conson

**Affiliations:** ^1^Department of Psychology, George Mason UniversityFairfax, VA, USA; ^2^Neuropsychology Laboratory, Department of Psychology, Second University of NaplesCaserta, Italy

**Keywords:** freezing, looming cognitive style, physical threats, anxiety, approaching motion

## Abstract

Immobilizing freezing responses are associated with anxiety and may be etiologically related to several anxiety disorders. Although recent studies have sought to investigate the underlying mechanisms in freezing responses that are so problematic in many forms of anxiety, cognitive factors related to anxiety have not been investigated. This study was designed to investigate the potential moderating role of a well-documented cognitive vulnerability to anxiety, the Looming Cognitive Style (i.e., LCS; Riskind et al., 2000), which assesses the extent to which individuals tend to routinely interpret ambiguous threats (e.g., physical or social threats) in a biased manner as approaching. We assessed participants' Reaction Times (RTs) when they made judgments about images of animals that differed in threat valence (threat or neutral) and motion direction (approach or recede). As expected, LCS for concerns about the approach of physical dangers appeared to moderate freeze reactions. Individuals who were high on this LCS factor tended to generally exhibit a freeze-response (slower RTs) and this was independent of the threat valence or motion direction of the animals. These general freezing reactions were in stark contrast to those of individuals who were low on the LCS factor for concerns about the approach of physical dangers. These participants tended to exhibit more selective and functional freezing responses that occurred only to threatening animals with approach motion; they did not exhibit freezing to neutral stimuli or any stimuli with receding motion. These findings did not appear to be explicable by a general slowing of RTs for the participants with high LCS. Moreover, the LCS factor for concerns about social threats (such as rejection or embarrassment) was not related to differences in freezing; there was also no additional relationship of freezing to behavioral inhibition scores on the Behavioral Inhibition System and the Behavioral Activation System Scales (BIS/BAS). It may prove fruitful to further explore cognitive factors related to anxiety to develop a more comprehensive understanding of how these factors are associated with anxiety-related freezing responses.

## Introduction

Approaching threats elicit defensive responses that had been shaped by natural selection. Such defensive responses have been found not only in human beings and their young, but also in other non-human vertebrate and invertebrate animals. Defensive responses have been broadly categorized into three types: freezing (to reduce an enemy's attention), fleeing (to increase the distance from the danger) and fighting (to dissuade the enemy; Blanchard et al., 1986; Eilam et al., 2011).

Freezing is a threat-related defense strategy characterized by a complete absence of movement, a tense body posture (with increased muscle tonus), a reduced heart rate (bradycardia); it represents an orienting response during which the animal is hypervigilant to cues priming an appropriate reaction, especially fight-or-flight behaviors (Hagenaars et al., 2014a; Mobbs et al., 2015). Recently, Gray and McNaughton (2000) have proposed the fight, flight, and freeze system mediates reactions to aversive stimuli and threats. The importance of freezing has also become increasingly more salient as it has been viewed as being involved in the etiology of threat-related disorders such as posttraumatic stress disorder (Hagenaars et al., 2008; Rizvi et al., 2008) and social phobia (Buss et al., 2004).

In humans, freeze-like behavior has been reported in previous studies (Carpenter et al., 1999; Azevedo et al., 2005; Roelofs et al., 2010; Hagenaars et al., 2012, 2014b; Ly et al., 2014), by assessing postural control and heart rate in response to aversive stimuli (e.g., unpleasant movies or angry faces). In particular, following animal studies Sagliano et al. (2014) specifically tested whether approaching motion of threatening stimuli can induce freezing (operationalized as a slowing of reaction times) in healthy humans as well. The authors used a modified version of the two-frame apparent motion paradigm, in which both size and location of a stimulus within a background were manipulated; by these means, the stimuli can be perceived approaching or receding. Participants had to perform a semantic decision task (living/not living judgments) and results showed that implicitly processed approaching threats (e.g., spiders or snakes) elicited a stronger freeze-like response with respect to receding threats. Findings from this study were in line with the hypothesis that freezing responses appear in dangerous context, as in the presence of an approaching threat, when individuals need to avoid being detected by the predator, have to optimize perceptual and attentional processes, and prepare the most useful responses (escape or fighting).

As suggested by Hagenaars et al. (2014a), some personal characteristics (e.g., behavioral inhibition and trait anxiety) seem to play a role in the selection of a particular defense response. Nevertheless, individual differences in freezing have rarely been studied. For instance, person's perceptions of personal vulnerability to dangerous situations could affect the duration and the presence of an adequate freezing response implies. This hypothesis is supported by previous studies showing that anxiety can modulate freezing responses in both animals (Frank et al., 2006) and humans (Roelofs et al., 2010; Sagliano et al., 2014).

Recent evidence from a different line of research has investigated an individual difference related to anxiety, called the Looming Cognitive Style (LCS), in the internalized tendency to overestimate the approach movement of threats (Riskind et al., 2000; Riskind et al., 2012). According to the model, approach movement is an intrinsic component of the threat value of potentially dangerous situations. The construct of LCS (Riskind et al., 2000) captures important aspects of a personal cognitive vulnerability or predisposition to anxiety. Individuals who have the LCS tend to habitually perceive and interpret ambiguous threats as rapidly approaching and escalating in risk, proximity and danger to themselves.

Accumulated research has shown that LCS is elevated in individuals with anxiety disorders (Riskind et al., 2011) and linked to a variety of processes and outcomes related to anxiety, including biased processing in threat-related memory and attention (Riskind et al., 2000), greater tendencies to overestimate the closeness of in approaching sound source (Riskind et al., 2013), greater reactivity to stressful life events (Adler and Strunk, 2010), and emotion dysregulation and fears of loss of emotional control (Riskind and Kleiman, 2012). LCS differs from anxiety sensitivity, worry, and intolerance of uncertainty (Riskind et al., 2000, 2007; Reardon and Williams, 2007) for it focuses on exaggerated perceptions of the approach movement of threat in time and space and its rapidly increasing proximity and intensity.

The LCS measure is divided into two subtypes (Riskind et al., 2000): physical looming, which refers to scenarios that are physically dangerous, and social looming, which pertains to mental simulation style for socially threatening scenarios. Despite the correlation of these two subscales, they can have their own divergent spheres of influence.

In the present study we focus on physical looming, which is the most relevant for the perception of physically threatening stimuli and, based on the foregoing considerations, we examined whether the extent that individuals have a characteristic looming cognitive style for physical danger modulates their freeze-like responses to approaching threats.

To verify this hypothesis, in the present study LCS for physical danger and freeze-like behavior were assessed in non-clinical individuals using the same methodology as in Sagliano et al. (2014). More precisely, participants were required to judge whether approaching or receding, threating and non-threatening, stimuli (animals and objects) were living or not-living. If freezing is a threat-related defense strategy preparing an appropriate reaction to an approaching danger, then we should find that individuals with Low Physical Looming (LPL) will exhibit a selective freeze-response to approaching threats that has ordinarily been found in prior studies. In contrast, we expect that individuals with High Physical Looming (HPL) have lost this adaptive behavior and will display a “dysfunctional freezing,” that is a generalized freeze-response to moving and in particular to approaching stimuli independently of their threat value (both threatening and non-threatening images). Their tendency to interpret ambiguous threats as approaching will induce more general freezing.

As a secondary goal, we also explored the potential effects of the LCS for social threat. We also assessed the potential contributions of participants' anxiety and of behavioral inhibition, which is a personality trait referring to a tendency to respond to threat with withdrawal (Hagenaars et al., 2014a). As suggested by Hagenaars et al., each of these individual differences might be related to freezing responses.

## Materials and methods

### Participants

One hundred right-handed, healthy undergraduate students (57 females; age range 20–33; mean age = 23, DS = 3.12) gave their written informed consent to take part in the experiment. Exclusion criteria were history of head injury, treatment with psychotropic medications, medical illness within 4 weeks before testing, self-reported mental or substance use disorder, current stressful episode or major life event.

All the participants completed the experimental tasks that had been previously approved by the local ethical committee (“Comitato Etico del Dipartimento di Psicologia della Seconda Università di Napoli”) and were conducted according to the Helsinki Declaration. Written informed consent was obtained from each participant involved in the study.

### Measures

#### Looming maladaptive style questionnaire

The Looming Maladaptive Style Questionnaire (LMSQ; Riskind et al., 2000; Italian version: Sica et al., 2012) is a well-validated 18-item scale evaluating the tendency to interpret and play out ambiguous threat situations in the mind as rapidly increasing in danger and moving closer in space and time (i.e., the Looming Cognitive Style: LCS). The person with this looming cognitive style perceives the chances of harm, the proximity of the harm, etc. increasing, escalating and becoming greater by the moment (or “looming”). For example, the participants are asked about a potential threat of an automobile accident “are the changes of your having an accident decreasing, or increasing and expanding with each moment?” And, “Is the level of threat staying fairly constant or is it growing rapidly larger with each moment.”

Participants respond to six vignettes describing a range of potentially stressful situations including physical health and injury (Physical Looming subscale) and social rejection (Social Looming subscale). Participants answer three questions about each vignette on a 5-point Likert-type scale. Individual item scores are aggregated such that higher scores indicate higher levels of looming vulnerability.

In the present study, the coefficient alpha was 0.86 for the Physical Looming subscale, 0.86 for the Social Looming subscale and 0.90 for the total scale.

#### Beck Anxiety Inventory

The Beck Anxiety Inventory (BAI; Beck et al., 1988) consists in 21-items assessing symptoms of anxiety occurring during the past week. Most items on this scale involve somatic indicators of anxiety. Questions were answered on a 4-point Likert scale ranging from 0 (“not at all”) to 3 (“severely—could barely stand it”). The alpha coefficient for the BAI was 0.91 in the present study.

#### Behavioral inhibition system and the behavioral activation system scales

The Behavioral Inhibition System (BIS) and the Behavioral Activation System (BAS) Scales (BIS/BAS; Carver and White, 1994; Italian version: Leone et al., 2001) consist of a 20 items self-report questionnaire designed to assess the responsiveness of Gray's (1982, 1987) BIS and BAS as personality characteristics. Questions were answered as one of 5 options, ranging from “Very false for me” to “Very true for me.” In the present study, the 7-items of the BIS scale were administered to evaluate the reactivity of the behavioral inhibition (or aversive motivational) system (scale range: 7–28); the Cronbach's alpha for the BIS scale was 0.83.

### Experimental task

Stimuli employed here were the same as those used in Sagliano et al.'s (2014) study. In particular, images were colored realistic pictures of 10 threatening animals (three species of spider, two species of scorpion, beetle, snake, crocodile, bee, angry dog) and 10 non-threatening animals (two species of rabbit, guinea pig, chick, cat, bird, duck, pig, tortoise, squirrel), and of 20 pictures of neutral objects (bottle, guitar, book, coffee maker, umbrella, ball, pot, camera, suitcase, mug, balance, kennel, sofa, motorbike, wall clock, pendulum-clock, piano, arm-chair, stool, table).

A preliminary assessment of the threatening valence of the experimental stimuli was conducted on a different group of subjects (*N* = 49). Stimuli were randomly presented in the center of the screen (in the same position and with the same size of the “medium-size stimulus” in the experimental task) on a white background and remained on view until participants' response. Participants were required to assess valence and arousal of each stimulus; they had to rate how unpleasant or pleasant each image made them feel on a 1–9 scale (1 = very unpleasant, 5 = neutral, 9 = very pleasant), and how emotionally aroused each image made them feel on a 1-9 scale (1 = calm, 5 = somewhat aroused, 9 = excited). Arousal and valence judgments were required randomly.

To confirm the differences in assumed threat valence, two *t*-test analyses were performed on valence and arousal judgments separately. Analyses showed that threatening stimuli achieved lower scores (mean = 3.30, SEM = 0.23) in valence judgements than non-threatening stimuli (mean = 6.59, SEM = 0.14), *t*_(92)_ = −11.27, *p* < 0.001; moreover, threatening stimuli were assigned significantly higher arousal ratings (mean = 6.40, SEM = 0.21) than non-threatening stimuli (mean = 2.49, SEM = 0.21), *t*_(22)_ = 12.26, *p* < 0.001.

**Figure 1:**
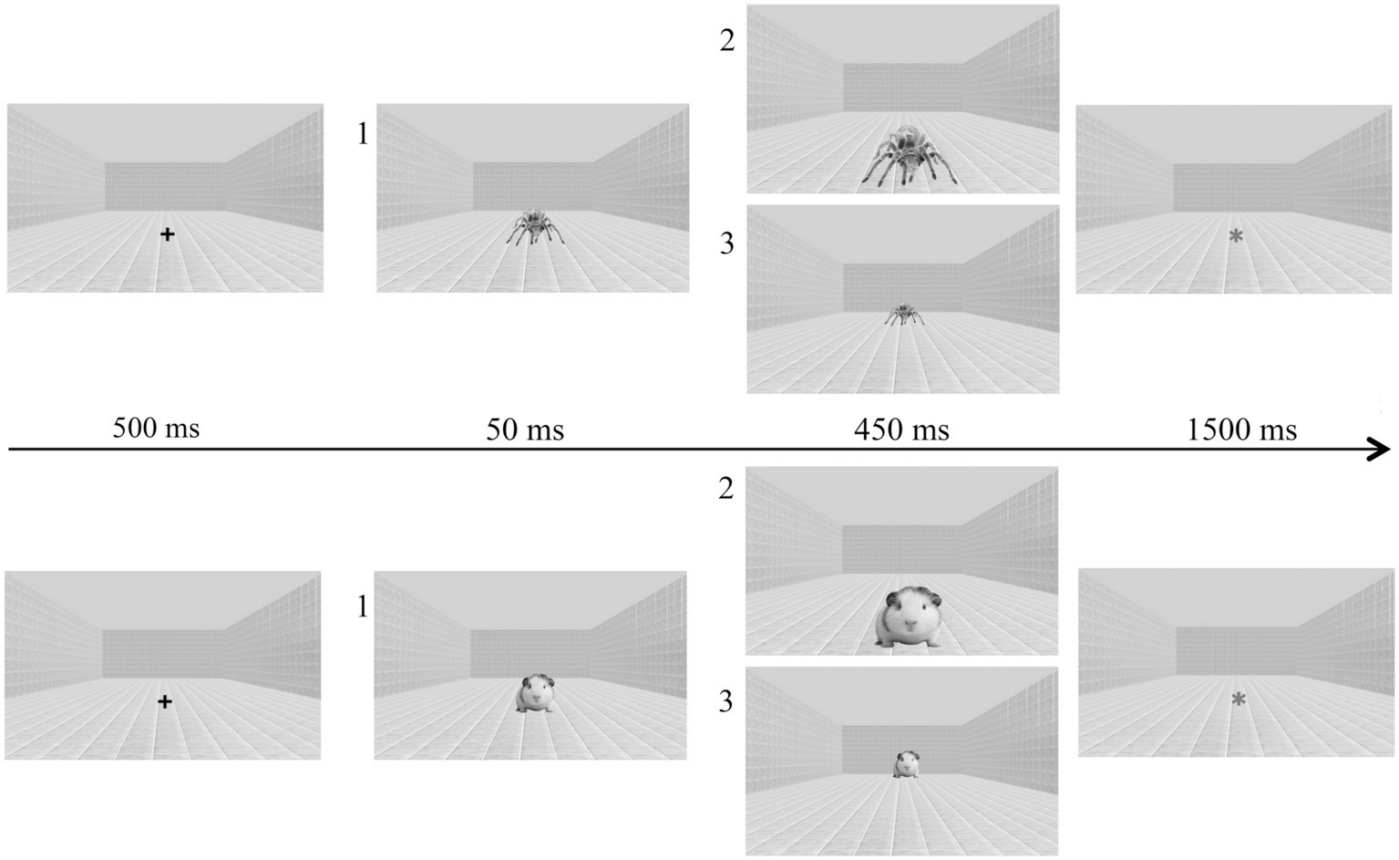
**Example of experimental stimuli (threatening: upper row; non-threatening: lower row) and of trial sequence**. Frame sequence 1–2 displays approaching stimuli, whereas frame sequence 1–3 displays receding stimuli. Here, images are presented in gray-scale, but they were employed in the natural colored version for the experiment. The trial sequence started with a fixation cross (+), followed by stimuli displaying an approaching or a receding motion. Finally, the stimulus was replaced by an asterisk (^*^).

In order to favor perception of depth, pictures were included in a background consisting of a room with gray-shaded tiled floor and walls in depth perspective. A modified two-frame apparent motion paradigm was used to produce a strong impression of stimulus motion (Sagliano et al., 2014). This paradigm implies that stimuli increasing in size radially outward from an unmoving center provide a visual signal of stimulus approach (Schiff et al., 1962). Thus, presenting two stimuli in rapid succession, differing only in size, apparent approaching (a small stimulus followed by a large stimulus) or receding (a large stimulus followed by a small stimulus) motion can be produced. To further enhance perception of motion we concurrently manipulated both stimulus size and location. Stimuli were displayed on a 19-in. computer screen (approximately 40 cm from the participant) at three different sizes: small (3.5 cm along the widest axis, 5° visual angle), medium (7 cm, 9.9°) and large (14 cm, 19.3°). Medium-size stimuli were always located in the center of the computer screen (position 1), large stimuli were presented 2 cm below the center of the monitor (position 2), and small stimuli 2 cm above the center (position 3; Figure 1). Presenting in rapid succession a medium stimulus in position 1 and then a large stimulus in position 2 the perception of approaching motion was triggered; presenting a medium stimulus in position 1 followed by a small stimulus in position 3 an apparent receding motion was elicited (Figure 1).

The task consisted in 160 trials: 20 neutral animals presented in approaching positions; 20 neutral animals presented in receding positions; 20 threatening animals presented in approaching positions; 20 threatening animals presented in receding positions; 40 neutral objects presented in approaching positions; 40 neutral objects presented in receding positions.

Each trial consisted of the following sequence of events: a fixation cross was shown for 500 ms in the center of the computer screen (position 1); then, in the same position the stimulus appeared for 50 ms, and was followed by the stimulus in position 2 or 3; after 450 ms, the picture was substituted for by an asterisk that remained on view until subject's response (Figure 1).

Participants seated with their head positioned on a chinrest and were instructed to keep their eyes on position 1 during the whole trial sequence. Participants were asked to judge whether the stimulus presented was living or not-living by providing their response when the asterisk appeared on the screen. The task consisted in two blocks: in one block, participants responded by pressing a right button for living-things and a left button for not-living things, whereas in the other block the response keys were inverted. All participants performed both blocks in randomized order. Moreover, they were instructed to provide their response as fast as possible. Reaction Times (RTs, ms) for living stimuli were recorded and submitted to statistical analysis whereas not-living stimuli were not included in the main data analysis, but were reserved for a secondary analysis to test for potential differences in overall processing speed by high and LCS Physical Looming participants.

### Procedure

All participants were asked first to respond to the Looming Maladaptive Style Questionnaire, the Beck Anxiety Inventory and the Behavioral Inhibition System Scale (measures were randomly administered to participants). Later, participants had to perform the experimental task.

## Results

As in a previous study (Sagliano et al., 2014), RTs data were cleaned first by removing trials in which participants made an incorrect response or did not respond, and then by removing RTs faster than 150 ms and slower than 1000 ms. After the cleaning procedure, data from 16 participants were not included in the analysis as they provided incorrect/no response or resulted in outliers (TRs < 150 or >1000) in about 30% of trials.

### Do anxiety and physical looming affect freeze-like response?

Mean anxiety and mean scores on the LCS-Physical factor for the entire sample were 14.32 (SEM = 1.16) and 32.06 (SEM = 0.68) respectively. Median splits were used to define participants into low anxious (*N* = 42) and high anxious (*N* = 42) and in Low Physical Looming (LPL; *N* = 41) and High Physical Looming (HPL; *N* = 43) groups (Descriptive statistics are reported in Table 1).

**Table 1:** **Mean, SEM and median of social looming subscale (SL), physical looming subscale (PL), looming maladaptive style questionnaire (LMSQ total score), Beck anxiety scale (BAI), and behavioral inhibition system (BIS) for LPL (Low Physical Looming) and HPL (High Physical Looming) individuals, and separately for Low and High anxiety level**.

	**LPL**		**HPL**	
	**Low anxiety**	**High anxiety**	**Low anxiety**	**High anxiety**
N	27	14	15	28
**SL**				
Mean	28.1	31.6	34.4	36.6
SEM	1.23	1.19	1.24	0.82
Median	27	30	35	36
**PL**				
Mean	27.1	26	36.3	37.6
SEM	0.73	0.91	0.84	0.67
Median	27	26	35	38
**LMSQ**				
Mean	55.2	57.6	70.7	74.2
SEM	1.57	1.74	1.73	1.24
Median	54	56.5	69	73.5
**BAI**				
Mean	6.8	20.1	8.1	23.9
SEM	0.69	2.55	0.89	1.81
Median	7	17	9	20.5
**BIS**				
Mean	22.6	23	23.1	23.5
SEM	0.58	0.75	0.56	0.71
Median	22	23.5	23	23

The first ANOVA was performed on RTs, with stimulus valence (threatening and non-threatening), motion direction (approaching and receding) as within-subject factors, and with the LCS-Physical factor (LPL and HPL) and anxiety (low and high anxiety) as between-subjects factors. A significant 3-way interaction was found among motion direction, valence and physical looming, *F*_(1, 80)_ = 6.50, *p* = 0.01, η^2^_*p*_ = 0.07. No main effect or other main effect or interaction for motion direction, the LCS-Physical factor, or anxiety was significant (all *p* > 0.05). When the 3-way interaction was decomposed by simple effects analyses, this revealed that there was a significant 2-way interaction between valence and physical looming for the approaching motion direction, *F*_(1, 80)_ = 11.02, *p* = 0.001, η^2^_*p*_ = 0.12, but there were no differences or effects for the receding motion direction (*p* > 0.5). Then, pairwise *t*-test comparisons on the significant 3-way interaction showed that the LPL group was slower in responding (freezing) to approaching threatening than to the other conditions (*p* < 0.05), whereas no significant difference among the four conditions was found in the HPL group (all *p* > 0.05; Table 2).

**Table 2:** **Mean RTs and SEM of LPL (Low Physical Looming) and HPL (High Physical Looming) individuals in the four experimental conditions (approaching/threatening, approaching/non-threatening, receding/threatening and receding/non-threatening)**.

		**LPL**		**HPL**	
		**Mean**	**SEM**	**Mean**	**SEM**
Approaching	Threatening	347.9	11.1	356.5	10.8
	Non-threatening	318.7	11.3	358.9	10.9
Receding	Threatening	332.4	11.2	355.3	10.9
	Non-threatening	332.2	11.8	354.2	11.5

**Figure 2:**
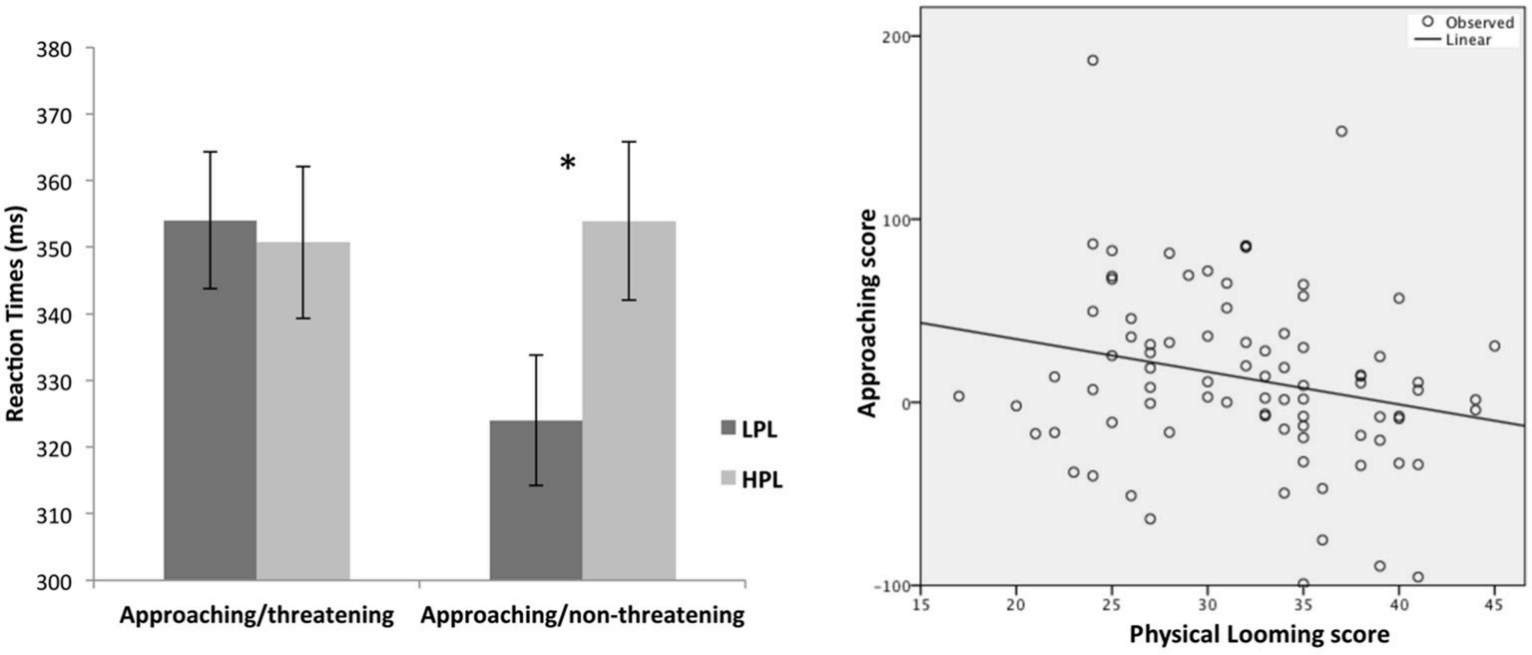
Left panel: RTs of LPL and HPL participants when judging approaching/threatening and approaching/non-threatening stimuli (^*^p = 0.05). Right panel: scatter plot showing the significant negative correlation between physical looming score and the “approaching score” when processing approaching threats (*p* = 0.035). A positive approaching score indicated a selective (functional) freezing to threatening stimuli, whereas scores close to 0, or even negative values, indicated a non-selective (dysfunctional) freezing response to both threatening and non-threatening stimuli.

As no simple effects differences were found between the LPL and HPL groups for the receding motion condition, we further performed independent samples *t*-test comparisons to specifically focus on group differences in responding to approaching stimuli. We compared RTs of two groups when judging approaching/threatening and approaching/non-threatening stimuli. Results showed that LPL and HPL participants did not differ when processing approaching/threatening stimuli (*p* > 0.05), whereas HPL were significantly slower than LPL participants in responding to approaching/non-threatening stimuli (*p* = 0.05; Figure 2).

In sum, these results demonstrated a selective (functional) freezing in the LPL group and a non-selective (generalized) freezing in the HPL group, indicating that the looming cognitive style moderated freezing responses on the task.

As yet another way to test the association between looming cognitive style and freezing responses, we computed an “approaching score” by subtracting RTs of non-threatening from RTs of the threatening trials: positive values indicated a selective (functional) freezing to the approaching threatening stimuli; values approximating to 0, or even negative values, indicated a non-selective (generalized) freezing to both threatening and non-threatening stimuli. Pearson's coefficient showed that the physical looming subscale score was negatively correlated with the “approaching score” (*r* = −0.230; *p* = 0.035; Figure 2). Thus, these data clearly demonstrate that freezing became more generalized and non-selective as scores on the LCS-Physical factor increased.

### Is freezing simply related to a general response slowing in the high physical LCS group?

As we indicated earlier, it is possible that the high LPL group's performance may simply reflected a general slowing response rather than impaired freezing. This would mean that the slower RTs to non-threatening animals (i.e., the apparent non-selective freezing) in HPL might merely be related to a general response slowing of this group. To assess this alternative explanation, we also analyzed RTs for not-living stimuli. The ANOVA with motion direction (approaching and receding) as within-subject factor, and with physical looming (LPL and HPL) and anxiety (low and high anxiety) as between-subjects factors did not reveal any significant main effect or interaction (for all factors, *p* > 0.05). That is, RTs of high and low PL participants did not significantly differ when non-living stimuli had to be judged. It would appear therefore that the alternative explanation is unlikely and our main results were not just due to response slowing.

### Is freeze-like response related to social looming?

Was freezing primarily associated with scores on the physical threat factor of LCS, or was it also related to scores on the social threat factor? To determine this, we conducted a separate analysis on the LCS-Social factor. Mean scores for the LCS-Social factor for the entire sample was 32.7 (SEM = 0.67). In order to verify whether freeze-like response to approaching threats was only affected by the LCS-Physical factor, the median of the LCS-Social factor was used to split the sample in two subgroup: low Social Looming (LSL; *N* = 42) and high Social Looming (HSL; *N* = 42).

An ANOVA was performed on RTs with stimulus valence (threatening and non-threatening), motion direction (approaching and receding) as within-subject factors, and social looming (LSL and HSL) as between-subjects factor. Results showed a significant 2-way interaction between valence and the LCS-Social factor, *F*_(1, 82)_ = 5.88, *p* = 0.02, η^2^_*p*_ = 0.07, as individuals with low scores on the social looming subscale were slower to respond to threatening stimuli (mean = 356.2, SEM = 10.13) compared to non-threatening stimuli (mean = 339.6, SEM = 10.5; *p* = 0.005). No main effect or other interaction was significant (all *p* > 0.05). We followed this up with an ANCOVA on RTs with stimulus valence (threatening and non-threatening) and motion direction (approaching and receding) as within-subject factors, with the LCS-Social factor (LSL and HSL) as the between-subjects factor and with the LCS-Physical factor as covariate. Results showed no significant main effect or interaction (all *p* > 0.05), thus implying that the significant 2-way interaction between valence and the LCS-Social factor was attributable to the correlation between the LCS-Social and Physical factors. On the other hand, controlling for social looming in an ANCOVA on RTs with stimulus valence (threatening and non-threatening) and motion direction (approaching and receding) as within-subject factors, with physical looming (LSL and HSL) as between-subjects factor and with social looming as covariate, did not alter the significant 3-way interaction described above among motion direction, valence and physical looming.

### Is freeze-like response related to the behavioral inhibition system?

The mean BIS subscale score for the entire sample was 23 (SEM = 0.34). To assess whether BIS scores were related to freeze-like responses the median of BIS scores was used to split the sample in two subgroups: low BIS (*N* = 38) and high BIS (*N* = 46). An exploratory ANOVA was performed on RTs with stimulus valence (threatening and non-threatening) and motion direction (approaching and receding) as within-subject factors, and with BIS group (low and high BIS score) as between-subjects factor. No main effect or interaction was significant (all *p* > 0.05).

## Discussion

It has been shown that individuals exhibit freeze-like responses to approaching threat, but individual differences in freezing responses have rarely been investigated (Hagenaars et al., 2014a). The present study is the first to demonstrate that such freezing depends on a cognitive vulnerability to anxiety. Specifically, we found that freezing responses are related to individual's habitual tendencies to perceive and interpret potentially threatening situations as rapidly approaching and rising in risk (i.e., LCS; Riskind et al., 2000). These findings are interesting because they are a step toward better understanding of individual differences that may play a role in anxiety-related disorders, such as PTSD (Hagenaars et al., 2008; Rizvi et al., 2008) and social phobia (Buss et al., 2004).

Specifically, we found an interaction effect indicating that the LCS for physical danger moderated the selective freezing response that was obtained in past studies. Previous researchers have conceptualized a selective and momentary freezing response (i.e., slower RTs) to images of approaching threatening animals (e.g., Sagliano et al., 2014) as being a functional adaptation because it allows for effective recruitment of resources in order to enhance perceptual and attentional processes to possible dangers (Kapp et al., 1992; Lang et al., 2000).

Such freezing can be an adaptive coping strategy when expressed in an appropriate context of actual danger, yet can become clearly maladaptive when sustained or expressed in inappropriate contexts since it hinders flexible responding to changes in the environment; when individuals are not able to show an adequate freezing response, in terms of both duration and context, they tend to remain immobile and vigilant irrespective of actual danger, thus limiting their ability to use adaptive coping strategies (Hagenaars et al., 2014a).

Consistent with our hypothesis that the LCS for physical danger is maladaptive in this context, the obtained interaction effect indicated that participants with high LCS for physical threat apparently lacked an adaptive or selectively “functional” freezing response: they showed freeze-like responses to the stimuli regardless of whether the stimuli involved threatening or non-threatening images. According to the looming vulnerability model, a person who has the physical danger component of LCS is more apt to automatically perceive and interpret ambiguous physical threats as threats that are approaching and escalating in physical danger (Riskind and Williams, 2006). Thus, such a person would be more likely to show freezing in inappropriate situations because of a more generalized tendency to detect and perceive ambiguous threats as approaching dangers. If so, such freezing reactions could hinder the person with the physical danger component of LCS from flexibly responding in changing environments and find effective coping responses.

Unlike Sagliano et al.'s (2014) study, which found that freeze-like responses were related to higher state anxiety as assessed by the State-Trait Anxiety Inventory (see also Roelofs et al., 2010), we presently found no effects for anxiety symptoms on the Beck Anxiety Inventory. The explanation for the differences in findings for anxiety needed to be further clarified, but could likely reflect the differences in the anxiety measures used.

It could be reasonably asked whether the present results merely reflect a generalized slowing of responses by individuals with high physical looming rather than a freeze response. For example, it might be possible that such individuals showed general slowing because they are ruminating or showing inhibition/interference problems. We found, however, that this is an unlikely explanation. First, we found a significant negative correlation of the physical looming subscale score with the “approaching score.” Second, we analyzed RTs for the non-living stimuli in order to test whether the high LCS participants (on the LCS-Physical Factor) were simply slower in general, rather than exhibiting freezing responses. Our analysis for the non-living stimuli seemed to rule this alternative explanation out because they showed that the freeze-like responses associated with the LCS were not related to a general response slowing.

We found that the moderating effects of the LCS were primarily due to the LCS-Physical Factor in the present study. Despite finding a significant interaction with the LCS-Social Factor, we found that this was erased when we controlled for the physical danger component of LCS. On the other hand, the effects of the physical danger component of LCS were not substantively changed when controlling for the social danger component of LCS. It appears likely that these findings may reflect the fact that the present experimental paradigm was more relevant to physical threats because the stimuli were images of physically threatening animals. Thus, in future research it will be instructive to study whether the social danger component of LCS, which is elevated in social anxiety and generalized anxiety disorders (Riskind et al., 2011), responds in any analogous way to social threat stimuli.

We also explored the effects of the BIS subscale, from the BIS/BAS, and found that BIS scores were not associated with freezing reaction. Of note, however, Gray and McNaughton's (2000) revised Reinforcement Sensitivity Theory now distinguishes a Fight-Flight-Freeze (FFF) system from the behavioral inhibition system. As the BIS/BAS was not designed to assess this FFF system, future research could investigate whether freezing responses are related to scores on a recently published self-report measure, the Fight-Flight-Freeze questionnaire (Maack et al., 2015).

Interestingly, Hong and Lee's (2015) recent study of the intolerance of uncertainty construct also suggests a connection between the LCS and freezing. LCS was found to be associated with to the “Inhibitory” component of the construct, which assesses paralysis or “freezing-up” under conditions of uncertainty, as well as delayed decision making and perseverative thinking about possible threats (Dugas et al., 2001; Dugas and Robichaud, 2007). But LCS was not significantly related to the Prospective component of intolerance of uncertainty, which represents a desire for predictability of future events and triggers engagement in strategies such as information seeking to reduce uncertainty.

The present findings contribute to accumulating research showing that the LCS is closely related to a variety of measures and measured correlates of anxiety, including worry and thought suppression. Moreover, LCS is elevated in patients with DSM-diagnosed anxiety disorders, and has been shown to predict cognitive biases (e.g., in memory for threatening material) associated with anxiety, and seems to function as a cognitive vulnerability that predicts future anxiety symptom changes and increases (Riskind et al., 2007; Adler and Strunk, 2010; González-Díez et al., 2015). Our results extend this large body of evidence and suggest that LCS may be associated with freeze-like responses to perceptions of threat in inappropriate contexts.

The present study has several limitations. As the study used an unselected sample of college students, caution is required in generalizing the present findings to actual anxiety disorder populations. In addition, Hagenaars et al. (2014b) have recently suggested that evidence of brachycardia is necessary to differentiate between defensive freezing responses and purely attention-related responses. Future research will therefore be needed to follow-up on the present findings using brachycardia or other measures to assess the correspondence among different measures of freezing. Future studies could also clarify the relationships between the LCS, the new FFF measure of freezing, intolerance of uncertainty, and behavioral outcomes such as those in the present experiment. Investigation of different anxiety disorders (e.g., simple phobias and social anxiety, panic disorder, etc.) would also be important in further research.

Despite its limitations, the present study takes an important first step and has several strengths. First, it used a previously validated behavioral outcome in a laboratory task and well-validated measures of LCS, anxiety, and BIS, and it had a reasonably robust sample size. Moreover, the study is novel as it provides the first interesting evidence to suggest that a cognitive vulnerability to anxiety (i.e., LCS) is linked to freezing responses. In light of the present findings, it may prove fruitful to further explore cognitive vulnerabilities to anxiety to develop a more comprehensive understanding of how such factors are related to freezing responses associated with anxiety.

## Author contributions

JR, LS, and MC conceived the study; JR, LS, LT, and MC designed the experiments; LS and MC performed experiments; JR, LS, and MC performed statistical analyses; JR, LS, LT, and MC wrote the paper.

### Conflict of interest statement

The authors declare that the research was conducted in the absence of any commercial or financial relationships that could be construed as a potential conflict of interest.
